# Motivations for entering and remaining in volunteer service: findings from a mixed-method survey among HIV caregivers in Zambia

**DOI:** 10.1186/s12960-015-0062-y

**Published:** 2015-09-02

**Authors:** Stephanie M Topp, Jessica E Price, Tina Nanyangwe-Moyo, Drosin M Mulenga, Mardieh L Dennis, Mathew M Ngunga

**Affiliations:** College of Public Health, Medical & Veterinary Sciences, James Cook University, Townsville, Australia; Centre for Infectious Disease Research, c/- PO Box 30388, Lusaka, Zambia; Nossal Institute for Global Health, University of Melbourne, Carlton, Australia; Population Council, One Dag Hammarskjold Plaza, New York, NY 10017 USA; Futures Group Global, c/- World Vision Zambia, Great East Rd., Lusaka, Zambia

**Keywords:** Zambia, Community health, Volunteers, Motivation, HIV

## Abstract

**Background:**

A high burden of HIV in many sub-Saharan African countries has triggered renewed interest in volunteer-based community health programmes as a way to support treatment roll-out and to deliver services to children orphaned due to HIV. This study was undertaken as an evaluation of a USAID project implemented by a consortium of 7 NGOs operating in 52 Zambian districts. We aimed to examine motivations for *becoming* volunteer caregivers, *experiences* in service and *commitment* to continue volunteering in the future.

**Methods:**

A mixed-method survey approach was adopted incorporating close- and open-ended questions. District selection (3 of 52) was purposive, based on representation of urban, peri-urban and rural volunteers from a mix of the consortium’s NGO affiliates. Individual volunteer recruitment was achieved via group information sessions and opportunistic sampling was used to reach a quota (~300) per study district. All participants provided written informed consent.

**Results:**

A total of 758 eligible caregivers were surveyed. Through parallel analyses of different data types and cross-over mixed analyses, we found shifting patterns in motivations across question type, question topic and question timing. In relation to motivations for entering service, responses to both open- and close-ended questions highlighted the importance of value-oriented functions and higher order social aspirations such as “helping society” or “humanity”. However, 70% of participants also agreed to at least one close-ended economic motivation statement and nearly a quarter (23%) agreed to all four. Illustrating economic need, as well as economic motivation, over half (53%) the study respondents agreed that they had become a volunteer because they needed help from the project. Volunteers with lower and mid-level standard-of-living scores were significantly more likely to agree with economic motivation statements.

**Conclusions:**

Reliance by national and international health programmes on volunteer workforces is rooted in the assumption that volunteers are less costly and thus more sustainable than maintaining a professional cadre of community health workers. Understanding individuals’ motivations for entering and remaining in volunteer service is therefore critical for programme planners and policy makers. This study demonstrated that volunteers had complex motivations for entering and continuing service, including “helping” and other pro-social values, but also manifest expectations of and need for material support. These findings contribute to evidence in support of various reforms needed to strengthen the viability and sustainability of volunteer-dependent services including the need to acknowledge and plan for the economic vulnerability of so-called volunteer recruits.

## Introduction

Since Alma Ata and before, community health worker programmes reliant on volunteer labour have been seen as a way to address issues of health care access and equity in low-income settings [1,2]. The recent introduction of HIV services in sub-Saharan Africa has been no exception. Over the past 15 years, the rapid scale-up of prevention and treatment programmes around the region [3-6] has resulted in a burgeoning of volunteer service-delivery programmes [7-13]. These programmes have focused on a range of clinical, palliative and social support services, such as medication dispensing, home-based counselling and testing and support for orphans and vulnerable children [9,14-17]. In the post-Alma Ata era, however, important unresolved issues concerning these programmes’ effectiveness, cost and sustainability have become the subject of debate [18,19]. Matters around selection, training and performance and the related role of volunteer motivation have become a current topic in the global health literature [20-23].

Volunteering and volunteerism have been defined in various ways but are typically understood to mean the devotion of energy and time to an activity or service without the expectation of financial reward [24]. In this study, we were interested in volunteerism as ongoing or “sustained helping without obligation” as defined by Omoto and Snyder [25]. This type of continuous, planned and unobligated helping is distinct from both “spontaneous helping”, which occurs in response to unanticipated incidents, and “obliged caregiving”, which implies a deeper social expectation to help, such as in the case of caring for a family member with a long-term illness [26].

Pinder defines work motivation as “a set of energetic forces that originates both within as well as beyond an individual’s being, to initiate work-related behaviour, and to determine its form, direction, intensity and duration” [27,28]. Understanding what motivates individuals to provide “unobligated” and “sustained” volunteer services has implications not only for programmes’ short-term success or failure but also for the longer term feasibility and cost-effectiveness of volunteer initiatives [26,29,30]. This is reflected in both historical and more contemporary debates around the tactics and ethics of volunteer recruitment and retention, especially in low- and middle-income settings [18,31]. Research in several low- or middle-income countries (LMIC) has noted that the hard economic realities experienced by volunteers in such settings create an often complicated interplay between individuals’ pro-social values on the one hand and the concurrent need to seek material compensation on the other hand [13,30,32-35]. A serious implication of such findings is that rather than being a strict matter of individual choice, volunteers may be entering and remaining in service because they lack alternative livelihood options [36,37].

Despite the recent proliferation of HIV-related volunteer programmes in sub-Saharan Africa, there remains comparatively little empiric research exploring the motivations of the individuals involved and the implications for their sustained service. Akintola [32] examined the motivations of 55 individuals from two home-based care organizations in South Africa, exploring perceived rewards from volunteering – such as acquiring skills and community recognition. The author proposed that volunteer programmes may be strengthened if these rewards were taken into account in the planning and management of the programmes. In a second study, Akintola [29] again probed the question of motivations among South African volunteers using functionalist theory and a qualitative approach to highlight the fact that many volunteers were unwilling or unable to continue volunteering for long periods without remuneration. In one of the few examples of quantitative work in this area, Maes [31] presented data from a random sample of 110 Ethiopian volunteers in order to examine the “average” motivational profile using a cultural consensus approach.

This work, notwithstanding, there remains insufficient research exploring and explaining the underlying motives and patterns of volunteerism in sub-Saharan Africa generally and among HIV caregivers specifically. Utilizing a mixed-method approach and conducted in a large sample of Zambian volunteer HIV caregivers, the present study was designed to identify and quantify factors related to individuals’ motivation for entering into and remaining in volunteer service.

## Methods

### Study context

Zambia, located in central sub-Saharan Africa, has a population of approximately 13 million people. Despite rapid economic growth in the past decade, 60% of the Zambian population lives below the poverty line and 42% are considered to be in extreme poverty [38]. Zambia has one of the highest rates of HIV in the world (14.3%) [39], and although per capita spending on health rose from USD 64 to USD 96 per person between 2006 and 2011, the health system remains severely undercapacitated. In 2010, the health care provider-to-population ratio was 107:100 000 as compared with WHO recommendations of 250:100 000 [40]. Severe human, financial and material resource shortages paired with Zambia’s high HIV burden have led to the emergence of various HIV volunteer caregiver programmes aimed at supplementing psycho-social, material and/or clinical support.

This study was undertaken as an evaluation of a USAID project implemented by a consortium of 7 NGOs operating in all 52 Zambian districts. At inception, all seven partners agreed upon a common set of basic care and support services that their volunteer caregivers would provide to persons living with HIV (PLHA) and to orphaned or vulnerable children (OVC). At the time of data collection for this study, which occurred in September and October 2012, the programme reported having approximately 30000 volunteer caregivers working nationwide.

The volunteer HIV caregiver project investigated in this study focused on the provision of psycho-social and palliative care, building upon and extending the work carried out by two prior volunteer projects, also funded by USAID. Reflecting the US government’s then-fresh emphasis under the Obama administration to promote greater country ownership [41], the grant included a mandate to transition all activities to local institutions. In light of this imperative, understanding the mix of motivations that influenced volunteers to enter and remain in service became a matter of pressing concern.

### Study aims and conceptual framework

This study aimed to advance understanding of why individuals in Zambia enter into and remain in volunteer service by identifying (a) motivations for volunteering (b) the extent to which these motivations are fulfilled in service and (c) factors related to discontinuation or prolongation of service.

In order to explore the motivations for entering and remaining in volunteer service, we adapted the functionalist approach used by Omoto and Snyder [25] and Clary et al. [26] among others. Functionalist theory suggests that thoughts, feelings and actions serve both personal and social “functions” that are important to an individual’s wellbeing. Omoto and Snyder [25] suggest that individuals volunteer in order to fulfil several of these underlying social and/or psychological functions, which can in turn be seen as proxies for motivations. These motivations, or mix of motivations, are central to both the initial act of volunteering and subsequent commitment to sustained service [26].

**Figure 1:**
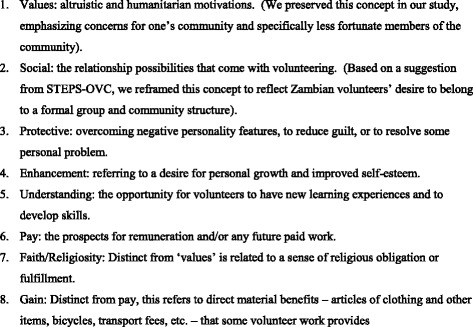
Definition of Theorized ‘Functions’ Selected for Inclusion in Initial Study Instrument.

From the literature, we considered the applicability of a range of theorized “functions” to the Zambian HIV caregiver context. Based on international and regional literature [24,29,31,36,37,42] and investigator experience in sub-Saharan Africa (SMT, JEP, TM, DM), an initial eight functions were proposed – values, social, protective, enhancement, understanding, pay, faith and other material gain. These functions were seen as crucial components of conceptual framework in which volunteerism was understood as a process of entering, experiencing and remaining in (or quitting) service. Our framework specified that the eight “functions” listed above were independent variables influencing entry to and expectations of service and interacted with contextual variables (for example household circumstances) and programmatic experience to influence commitment. A definition of each of the functions is outlined in Figure 1.

### Study and instrument design

We used a fully mixed-method survey approach [43] incorporating qualitative and quantitative methods applied sequentially and concurrently. An initial survey tool was developed in English based on the functions identified above. Following recommended practice for cross-cultural research [44,45], translation from English into three local languages – Nyanja, Bemba and Tonga – involved a question-by-question review in focus group discussions with eight active volunteers led by study staff. This was followed by formal pretesting and back translation to English.

To determine equivalence between the 3 study languages and with intended meanings in English, as well as to assess contextual relevance and effectiveness of specific questions, we conducted a validation study with a sample of 200 volunteers working under the same USAID project and representing all 3 language groups; these respondents were subsequently excluded from eligibility in the main study. The validation study results revealed the need for several modifications to the initial instruments. Primary among these were confusions arising from the use of Likert scales that proved both conceptually and linguistically unwieldy. Multiple versions and formats of Likert-like scales on fixed-choice questions (5-point, 7-point and 10-point; verbal and visual) proved ineffective in pretesting and in the validation study. As a result, questions related to motivations for becoming a volunteer and remaining in service were modified to include dichotomous response options such as “yes”/“no” or “agree”/“disagree”. Additionally, responses to two questions asking volunteers to estimate their time in service, an important potential variable, proved highly inconsistent and unreliable resulting in their omission from the final instrument. A multi-lingual team re-examined each question for linguistic, semantic and contextual equivalence and recommended translations to the final instrument used in the main study.

The final instrument included 49 fixed-choice and 7 open-ended questions (Table 1). Corresponding with the objectives of this study, the survey was structured to assess the following: i) demographic information and standard of living data, ii) motivations for becoming a volunteer caregiver, iii) experience in volunteer service and iv) motivations to continue volunteering. Explanation of the questions included in each section is provided below.

**Table 1:** **Instrument questions and key study concepts and measures**

**The volunteer process**				
**Motivations to enter service**		**Experiences in service**		**Commitment**
***I wanted to become a volunteer because/to…***		***List three things that prevent you from carrying out your caregiving work.***		***Do you intend to continue volunteering?***
1 = agree or 0 = disagree				1 = yes or 0 = no
Communitarian values		I fail to visit my clients because I…		
1	People need a helping hand and I felt obligated	1 = sometimes or frequently or 0 = rarely or never		List three things that would make you willing and able to perform more volunteer care giving in the future.
2	I saw too much suffering in the community			
Religiosity		1	Do not have transport	
3	People who believe in God should volunteer	2	Felt bad about going empty handed	
4	I would be doing the work of God	3	Lacked supplies and kits to do the job	
Social change agent		4	Never receive money for the work	
5	I wanted to change bad behaviours in the community	5	Do not have enough food or money at my own home	
6	Protect the rights of OVC and people with HIV	6	Am sick	OVC visitation rate:
Learning opportunity		7	Do not have rain gear	Number clients visits last month/
7	I wanted to learn new things	8	People think I keep things provided by the project for myself instead of giving them to clients	Total number clients assigned
8	Learn about HIV and how to take care of people			
Empathy, reciprocity				PLHA visitation rate:
9	I empathize with people in the same situation as me	Open ended: Thinking about why you first wanted to become a volunteer, how have your expectations been met?		Total number clients visited last month/
10	A volunteer helped me and wanted to give back			total number clients assigned
Social engagement				
11	Be part of a project	Open ended: How have your expectations not been met?		
12	I have friends and family who volunteer			
Material gain, support from the project				
13	I needed assistance from the NGO/project			
14	Receive things, allowances to help in my household			
Employment potential				
15	I thought it may channel me to a paying job			
16	I have no job			
Open ended: If you had to pick just one reason why you wanted to become a volunteer, what would you say it is?				
**Economic motivation index**				
Empirically derived 11-point index reflecting the presence or absence of economic motive in respondent replies (see Table 2)				

Demographic information and standard of living were assessed using household standard of living (SOL) and was calculated based on nine proxy indicators including access to improved water and sanitation and type of roofing and flooring materials [46]. An ordinal SOL variable was generated ranging from 0 (lowest) to 9 (highest). We defined value ranges of 0–3, 4–6 and 7–9 as “low”, “mid-range” and “high” SOL, respectively.

Motivation to enter service (column 1, Table 1) was assessed through 16 fixed-choice statements and 1 open-ended question. Mixed questions were also used to assess experience in service (column 2, Table 1) by examining barriers to caregiving and expectations met and not met during volunteer service.

To assess motivation to continue volunteering, we initially used “intent to continue” volunteering as the key measure of commitment to service. However, during the validation study, multiple questions repeatedly failed to produce any variability in the responses, with virtually 100% of the respondents indicating their intention to continue volunteering in the future. As an indicator of commitment to service, we therefore replaced “intent to continue” with a measure of volunteer performance. Project standards specify that beneficiaries should receive at least one home visit per month, so we considered visitation rates (number visited last month/number assigned) as demonstrative of volunteer commitment. In the final version of the instrument, we thus asked individuals to indicate the number of each type of beneficiary – OVC and PLHA – currently assigned to them and, of these, how many were visited last month (column 3, Table 1). These measures were supplemented with an open-ended question to probe individuals’ motivations to continue and to increase their volunteer work in the future.

### Sampling and fieldwork procedures

The sampling approach for this study was influenced by the geographic, organizational and linguistic spread of the volunteer programme under study. Lack of a central volunteer roster as well as inevitable resource constraints made randomized selection based on geographic location or organizational affiliation unfeasible. Instead, 3 out of the 52 districts were purposively selected based on representation of urban, peri-urban and rural sites and to ensure representation of caregivers working under all the NGOs affiliated with the project. Based on resource availability, we adopted a nominal sampling quota of 300 volunteers per district. Since individual tracing of volunteers to their homes was not feasible, recruitment was achieved by working directly with project coordinators to announce the study through group meetings and invite participation on subsequent nominated interview days at each site.

Volunteers who were ≥18 years old (a requirement of the respective ethics committees consulted) and recognized by the project as active for ≥6 months were eligible to participate. Although limiting our ability to understand the motivations of new recruits, the latter eligibility criteria were in line with one of the study’s primary objectives, which was to understand volunteers’ experience in service as part of this particular USAID programme. All eligible volunteers presenting on pre-announced interview days and who provided their informed consent to participate in the study were interviewed. Interviewing stopped once district quotas were met or the fieldwork schedule came to an end, whichever came first.

Interviewers were equipped with printed paper question guides in Nyanja, Bemba, Tonga and English. Interviews were conducted according to the participants’ language preferences. Logistical constraints made recording and transcription unfeasible for a sample size this large. To facilitate later transcription, interviewers thus translated and transcribed in English participants' replies to the short-answer open-ended questions on the paper survey tool. Prior to each volunteer participant leaving, field team supervisors conducted quality control checks to identify data inconsistencies and review clarity and legibility of written responses.

### Data preparation and analysis

Fixed-choice data were entered manually into SPSS Version 20.0 (IBM Corp, 2011) and later verified consecutively by two data entry clerks. Open-ended replies were transcribed in full and uploaded to NVivo Version 10 (QSR International Pty Ltd., 2012) as free text. We conducted both “parallel” analysis (independent analysis of the two types of data) and “cross-over mixed analysis” [47] (transformation of free text generated by open-ended questions into numeric variables, enabling integration with, and comparison to, quantified responses from the close-ended questions).

**Figure 2:**
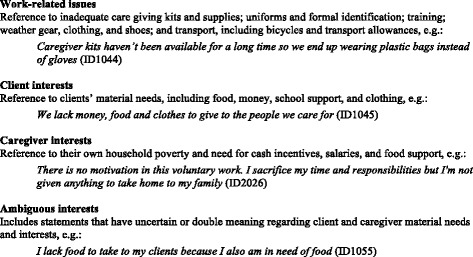
Main barriers and expectations-not-met themes.

Free-text data was reviewed to devise an initial coding scheme. This was further elaborated and refined through team-based, collaborative coding which made the task of managing 758 transcripts efficient while helping to ensure coding consistency. To facilitate analysis of free text, text data were then “quantitized” [48]. For this purpose, we converted qualitative themes into numeric variables with dichotomous values indicating the presence (=1) or absence (=0) of coded text for each respondent. The resultant participant-by-code matrix facilitated aggregate recoding of text units into increasingly inclusive thematic clusters, for example, “lack of transportation allowance” and “lack of a bicycle” codes were collapsed into “transport” and, in turn, into “work-related issues”. Quantitized free text also allowed us to consolidate the two data types into one quantitative integrated dataset thereby facilitating within-case and cross-over mixed analyses. Given the large amount of free-text data, coding reliability checks were carried out only on the broadest themes of interest. For each case, JP independently coded the presence or absence of the main analytic themes described in Figure 2 in the “Findings” section. SMT and JP reviewed each discrepancy found and came to consensus on the proper coding.

Statistical procedures were performed in SPSS. Descriptive analysis allowed us to determine the prevalence of themes, thoughts and experiences in the population across different questions and variables. To compare groups of nominal variables, we used Pearson’s chi-square test. On ordinal/interval variables, differences between groups were analysed using Mann–Whitney *U* test and Kruskal–Wallis *H* test, respectively, for two and three group comparisons. Correlations between ordinal/interval variables were tested with Spearman’s rho.

Early on in the analysis, we observed inconsistencies within individuals’ replies concerning their motivations based on economic need or desire for monetary payment or some other material reward for their work. To account for this within-case complexity, we developed an empirically derived “Economic Motivation” index to reflect the consistency of individuals’ expression of economic motivation across the entire interview (bottom row, Table 1). The 11-point index (0 to 10) is based on within-case analysis using responses to fixed-choice items and dichotomously coded free text to signify the presence (=1) or absence (=0) of economic motivation in each reply. The summed total indicates the least (0) and most (10) economic motivation. Table 2 details the within-case analytic procedure used.

**Table 2:** **Economic motivation index (economic motivation indicated by the presence of caregiver or ambiguous interest themes)**

**Interview sections and questions**		**Index values**
Motivations for becoming a volunteer		
	I wanted to become a volunteer…	
1	Because I needed assistance from the NGO/project	Agree = 1 Disagree = 0
2	To receive things, allowances to help in my household	
3	Because I thought it may channel me to a paying job	
4	If you had to pick just one reason why you wanted to become a volunteer, what would you say it is?	Economic motivation: Cited = 1 Not cited = 0
Experience in service		
5	List three things that prevent you from carrying out your care giving work	Economic motivation: Cited = 1Not cited = 0
	I fail to visit my clients because…	
6	I never receive money for the work	Sometimes or frequently = 1
7	I do not have enough food or money at my own home	Rarely or never = 0
8	Thinking about why you wanted to become a volunteer, how have your expectations been met?	Economic motivation:
		Cited = 1
9	How have your expectations not been met?	Not cited = 0
Motivations to continue volunteering		
10	List three things that would make you willing and able to perform more volunteer care giving in the future	Economic motivation:
		Cited = 1
		Not cited = 0
0–10		

## Ethics

The field supervisor first explained the study to the whole group of volunteers present at the study site. This included reviewing the study risks and benefits and responding to the volunteers’ questions. Interviewers also explained the study to individual participants and obtained their written informed consent before starting the interview. Permission to conduct this study was obtained from the University of Zambia’s Biomedical Research Ethics Committee, and a waiver was granted from the Population Council’s Institutional Review Board.

## Findings

Sample characteristics: a total of 802 individuals were interviewed for the study. Forty-four cases were excluded having not met inclusion criteria. The remaining 758 individuals constitute the evaluable population analysed in this paper. Table 3 summarizes demographic findings. Females had fewer years of education compared to males (*P* ˂ .001), and no difference in SOL was found. Compared to urban residents, rural residents reported fewer years of education (*P* ˂ .001) and lower SOL (*P* ˂ .001). Whereas 27% of female respondents were widowed, less than 1% of men were widowed. All but 5 volunteers reported having between 1 and 55 OVC clients assigned to them for an average of 9.8 per volunteer. Eighty-six per cent reported having between 1 and 50 PLHA clients with an average of 5.8 per volunteer. In relation to OVC and PLHA clients, respectively, we found mean visitation rates of .80 (SD = .30) and .84 (SD = .31).

**Table 3:** **Sample characteristics**

				***N***
Gender				
Male	Female			
304 (40%)	454 (60%)			758 (100%)
Residence				
Lusaka	Chongwe	Mpika		
406 (54%)	186 (24%)	166 (22%)		758 (100%)
Age				
Mean (SD): 43 (11.7)				756 (99.7%)^a^
Median: 43				
Min–Max: 18–81				
Marital status				
Married/cohabiting	Divorced/separated	Widowed	Never married	
505 (67%)	55 (7%)	153 (20%)	45 (6%)	758 (100%)
Education				
Mean (SD): 8 (3.6)				758 (100%)
Median: 8				
Min–Max: 0–18				
Standard of living (SOL) (9 household indicators)				
Mean (SD): 4.4 (1.85)				736 (97%)^b^
Median: 4				
Min–Max: 1–9				

^a^Excludes two individuals who did not know their age.

^b^Excludes 22 cases with missing data on 1 or more SOL items.

### Variables associated with client visitation

Overall, OVC and PLHA client visitation rates were .80 and .84, respectively. Women reported significantly higher client visitation rates compared to men, for both OVC (*P* = .036) and PLHA (*P* = .01) clients. No difference in client visitation was found between urban and rural residents or between SOL categories (Table 4). We found no correlation between economic motivation index values and OVC visitation rate (*r*_*s*_ = −.063, *P* > .05); similarly, no correlation was found between economic motivation and PLHA visitation rate (*r*_*s*_ = −.065, *P* > .05).

**Table 4:** **Motivations, barriers and client visitation rates by gender, residence and standard of living**

	**Gender**			**Residence**			**Standard of living**			
	**Male**	**Female**	**sig**	**Urban**	**Rural**	**sig**	**Low**	**Mid-**	**High**	**sig**
	**(** ***n*** **= 184)**	**(** ***n*** **= 574)**		**(** ***n*** **= 454)**	**(** ***n*** **= 304)**		**(** ***n*** **= 273)**	**(** ***n*** **= 340)**	**(** ***n*** **= 123)**	
“Agree”										
I wanted to become a volunteer…^a^										
To give back	63%	65%	--	63%	66%	--	65%	66%	55%	--
To be part of a project	77%	83%	--	82%	81%	--	79%	84%	77%	--
Friends and family volunteer	64%	65%	--	63%	68%	--	69%	66%	55%	.019
Needed project assistance	47%	54%	--	51%	55%	--	57%	54%	35%	˂.001
To receive things, allowances	26%	40%	˂.001	40%	32%	--	39%	37%	34%	--
To channel me to a paying job	30%	47%	˂.001	48%	35%	˂.001	44%	46%	41%	.049
I have no job	31%	51%	˂.001	46%	46%	--	53%	46%	29%	˂.001
“Sometimes or frequently”										
I fail to visit my clients…^a^										
Do not have transport	26%	28%	--	29%	26%	--	29%	29%	26%	--
Feel bad going empty handed	33%	43%	.02	47%	31%	˂.001	37%	43%	46%	--
Lacked supplies and kits	32%	39%	--	41%	33%	.033	33%	39%	48%	.017
Never receive money for the work	7%	11%	--	12%	6%	.013	12%	9%	8%	--
Not enough food or money at home	8%	12%	--	13%	8%	.04	14%	10%	10%	--
Am sick	3%	2%	--	2%	2%	--	2%	2%	5%	--
Do not have rain gear	35%	50%	--	43%	32%	.002	40%	37%	42%	--
People think I keep clients’ things	11%	22%	.001	26%	10%	˂.001	15%	23%	24%	.048
Economic motivation index	$\overline{x}$ = 2.21	$\overline{x}$ = 2.93	˂.001^b^	$\overline{x}$ = 3.06	$\overline{x}$ = 2.29	˂.001^b^	$\overline{x}$ = 2.84	$\overline{x}$ = 2.84	$\overline{x}$ = 2.48	--^c^
	*s* = 1.78	*s* = 2.01		*s* = 2.06	*s* = 1.76		*s* = 1.99	*s* = 1.95	*s* = 2.06	
OVC client visitation rate	$\overline{x}$ = .75	$\overline{x}$ = .81	.036^b^	$\overline{x}$ = .78	$\overline{x}$ = .82	--^b^	$\overline{x}$ = .82	$\overline{x}$ = .80	$\overline{x}$ = .73	--^c^
	*s* = .30	*s* = .30		*s* = .31	*s* = .29		*s* = .26	*s* = .33	*s* = .32	
PLHA client visitation rate	$\overline{x}$ = .81	$\overline{x}$ = .88	.01^b^	$\overline{x}$ = .86	$\overline{x}$ = .88	--^b^	$\overline{x}$ = .88	$\overline{x}$ = .86	$\overline{x}$ = .86	--^c^
	*s* = .25	*s* = .28		*s* = .31	*s* = .21		*s* = .28	*s* = .26	*s* = .30	

^a^Pearson chi-square test.

^b^Mann–Whitney *U* test.

^c^Kuskal–Wallis *H* test.

### Motivations for becoming a volunteer

Values-oriented and helping themes dominated responses to open-ended questions about motivations for becoming a volunteer caregiver. Typically expressed as personal characteristics (“I love to help others”, ID1004), communitarian goals (“I wanted to bring development to my community”, ID2151) and religious values (“We are taught as Christians to look after the needy”, ID1267), helping themes were cited by 90% of the respondents. Not surprisingly, HIV-related motives were prominent, framed variously as higher order aspirations – such as “saving humanity [from HIV]” (ID3086) and “helping my country progress by reducing HIV” (ID2088) – to much more precise motives to promote testing, provide support to OVC and PLHA, reduce stigma and promote behaviour change.

In response to close-ended questions about motivations for becoming a volunteer, we found almost universal agreement (94–99%) with motivation statements focused on helping values and learning opportunity (items 1–9 in Table 1). However, responses to close-ended statements focused on potential social and material advantage from volunteering generated more differentiated responses. In contrast to qualitative findings, 70% of respondents agreed to at least one of the economic motivation statement (items 4–7, Table 4) and 23% agreed to all four. Compared to men, women were significantly more likely to agree that they became a volunteer “to receive things and allowances from the project” (*P* ˂ .001), “to get a paying job” (*P* ˂ .001) and “because I have no job” (*P* ˂ .001). Notably, 53% (*n* = 398) of all respondents agreed to the statement, “I wanted to become a volunteer because I needed help from the project”. Individuals in low- and mid-level SOL categories were significantly more likely to agree with this statement compared to those in the high SOL category (*P* ˂ .001).

### Expectations met

We found a number of thematic similarities in responses to questions about whether expectations of volunteer service had been met. Personal values (“I feel sanctified because I’m doing the work of God”, ID1100), the ability to help others (“I’ve managed to get HIV-positive people on treatment and out of depression”, ID1324) and contributing to bettering their communities (“Stigma has become a thing of the past”, ID1082) were all frequently cited themes and indicated as expectations met by 36%, 33% and 27% of the respondents, respectively.

Eighty-five (11%) individuals indicated a desire to learn as a main motivation for entering service, and many more (32%) cited learning and personal growth as an expectation met from the experience. Learning skills to take care of an HIV-infected family member or oneself was cited as a motivation to become a volunteer by 55 (7%) individuals.

Forty respondents (5%) indicated economic motivation for becoming a volunteer, and most who did described their own beneficiary-like need as a justification:

I am in so much poverty. I joined so that I could be helped as well (ID1290)

On my own I can’t make it in life. I volunteered so I could meet people who can help me (ID1106)

Being a widow I thought that if I offer my services I’d get something, like a monthly allowance, to assist me and my family (ID1164)

### Barriers to service delivery and expectations *not* met

In response to close-ended questions, 67% of respondents indicated “sometimes or frequently” failing to visit clients in response to at least one failure-to-visit statement, and 364 (48%) selected this response option on two or more of the statements. On several statements, urban residents were more likely to respond that they “sometimes or frequently” failed to visit clients by comparison to rural residents (*P* ˂ .05) (Table 4).

Substantial thematic overlap was found in the responses to open-ended questions on barriers to caregiving and expectations not met. Free text from the two expectations not met questions generated 30 unique codes that were successively compressed and ultimately assigned to 1 of 4 broad themes: (i) work-related issues, (ii) client interests, (iii) caregiver interests and (iv) ambiguous interests (confounding the caregivers’ personal needs with their clients’ needs). These four themes are defined in Figure 2.

The most frequently cited barriers, work-related issues, were relatively straight forward. Lack of transportation to make home visits or to transport clients to health facilities were by far the most frequently cited barriers (73%), followed by incomplete or unavailable homecare kits and supplies (35%) and lack of weather gear, clothing and shoes (22%). Thirty-two per cent of respondents described similar work-related complaints as expectations not met by the volunteer programme.

Rather more complex were the volunteers’ descriptions of clients’ as well as their own material needs. The inability to assist beneficiaries materially was cited as a barrier to caregiving by 60% of the respondents and indicated as an expectation not met by 33%. Illustrated in the quotes below, these client-focused issues produced feelings of shame, ineffectiveness and low morale:

I feel ashamed visiting clients knowing that they don’t have anything to eat (ID1251)

I feel bad, like I’m not doing anything to better their lives (ID1969)

Because I keep visiting clients empty handed and only offer counseling, I feel discouraged and unmotivated (ID1697)

Feelings of shame and discouragement were compounded by beneficiary expectations of the project and of the caregivers themselves:

Clients want food and clothes. They complain a lot, but we have no means of helping them with these things (ID1104)

Stressing that “health talks” and “psycho-social support” were not enough, a number of respondents described how they became subject to suspicion and accusation when they failed to deliver on their clients’ expectations of material support:

My clients think I take their money or food that comes from the project (ID1229)

Clients chase us away because they think we eat the food from the project that is intended for them (ID1137)

One hundred and fifty-two (20%) volunteers indicated that failing to provide their clients with material support led to negative experiences. Beneficiaries “hiding”, being “hidden by relatives” or “running away” from “empty handed” caregivers were the most common negative experiences reported, although more dramatic encounters were also described:

Once I took a blouse [to a client] and she threw it back at me, yelling that she can’t eat a blouse (ID1053)

The sense of distrust expressed by the beneficiaries also appeared to influence the volunteers’ trust in the programme, forcefully expressed by this 52-year-old woman: “Sometimes I feel like a liar. At the beginning we told the children and their guardians that they will be given food and other things, but these things have never come” (ID1099).

The volunteers’ own economic need was cited as an obstacle to carrying out caregiving work by 182 (24%) individuals, and similar needs were described as expectations not met by the project by 121 (16%) individuals. The obligation to provide for their own families dominated these responses:

Being a bread winner it’s difficult to split my time fending for my own family and volunteering. The only ones eligible to receive food and other materials are the OVCs, not our own children, so it gets de-motivating sometimes (ID3135)

Sometimes we get food stuffs, like mealie meal, and our clients get everything. We caregivers have nothing, yet we also need this food but cannot beg from our clients (ID1218)

I fail to divide myself when it comes to looking after my family and the clients (ID1107)

The caregivers’ own household food insecurity was also explicitly cited as a barrier, an expectation not met, or both by 59 (8%) individuals: “It’s hard to work for another person when there is hunger at home” (ID2077).

In the free-text data on barriers and expectations not met, ambiguity between the economic interests of the volunteers and of their clients also begins to emerged. Responses in which volunteers linked their own economic wellbeing to that of their clients were included in this ambiguous interest theme.

I don’t have money to give my clients because I don’t work and I don’t have a business (ID1080)

The lack of income earning activities is a challenge for us as we don’t have money to buy food for our clients or for our own children (ID1306).

Two hundred and ten (28%) individuals gave ambiguous replies to the barriers question, to the expectations not met question or to both.

### Motivations to continue

In response to the final question of the interview – “List three things that would make you willing and able to perform more volunteer care giving in the future” – we found familiar themes, but with the exception of work-related issues, these themes were patterned differently. Whereas fewer individuals (41%) cited client interests compared to responses from questions posed earlier in the interview, more volunteers cited caregiver and ambiguous interests. For example, 46% of individuals indicated their own economic need in their replies:

We need to be employed like other workers (ID2008) and if I’m put on salary I’ll work harder (ID1118)

Caregivers should be provided incentives so our children don’t starve to death while we visit our clients (ID1117)

If I’m given motivational support, which can be either monetary or material (ID1024)

Cited by 33% of participants, ambiguous interests (that is, interests that appear to confound the caregivers’ personal needs with their clients’ needs) were more diversely articulated. One prominent ambiguous form that we identified in the motivation-to-continue question included “enable me to help my client”, for example:

As caregivers we should be empowered with income generation activities so that we are able, in turn, to look after our clients (ID1033)

I want to receive help with my farm so that I can take food to the sick (ID3108)

A variation on this form were “enable me to help myself and my client” statements. In these replies, the commonality of material need of caregiver and client was fully acknowledged:

We need start-up income, like from a tailoring business, so we can sustain ourselves, our families and our patients (ID1361).

If we were given fertilizer we could farm our own food and that way also help our clients better (ID2034)

The same basic form and variant were found in other replies but were expressed in a way that depicted a more direct role of volunteers as conduits of material support between the project and the clients. Examples of this “give me to give my client” form include the following:

If I’m provided money to give to my clients (ID1112)

Provision of money for me and my clients (ID1134)

Desire to have beneficiary status or to receive beneficiary advantages were also coded to the ambiguous interest category, for example:

If I can also benefit from the things that we give our clients, after everyone has gotten their share, this will motivate me (ID1339)

Regardless of how the volunteers replied to questions about their motivations for entering and staying in service and about their experiences in service, all 758 individuals replied “yes” to our fixed-choice screening question: “Do you intend to continue volunteering in the future?”

### Cross-method analysis

**Figure 3:**
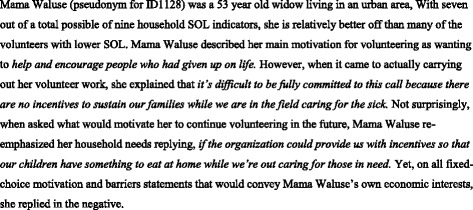
Mama Waluse – an example of overlapping motivations.

At the level of the population, the findings were fairly consistent: values-based and *other-*oriented themes were expressed as motivations for becoming a volunteer and as expectations met from the programme. Personal economic needs and material *self-*interest were, on the other hand, more often expressed as work barriers and as expectations not met. At the level of the individual, however, the *other*- versus *self*-oriented findings were often in tension. The example of “Mama Waluse” (Figure 3) is illustrative of this tension. Understanding the multiplicity of motivations and interests such as Mama Waluse’s in a coherent manner presents an obvious methodological challenge. The economic motivation index described in Table 2 was developed to confront this challenge.

Table 5 shows the frequency distribution of 0 to 10 economic motivation index values in the population. Of the 758 individuals in our sample, 12% (*n* = 91) had an index value of 0 (zero), meaning that they did not indicate economic motivation at any point in the interview. However, the remainder of the volunteers had values of at least 1, and 378 (50%) individuals showed values of 3 or higher. Consistent with findings on individual variables, women expressed significantly more economic motivation compared to men (*P* ˂ .001) as did urban compared to rural residents (*P* ˂ .001). There was no apparent correlation (*r*_*s*_ = −.052) between SOL and economic motivation index values (*P* > .05) nor were differences found in economic motivation between SOL categories (Table 4).

**Table 5:** **Frequency of economic motivation index values**

**Index value**	***N***	**%**
0	91	12.0
1	158	20.8
2	131	17.3
3	117	15.4
4	113	14.9
5	69	9.1
6	47	6.2
7	21	2.8
8	10	1.3
9	1	0.1
10	0	0
	758	100

## Discussion

This study adds to a small body of empiric research on volunteers and volunteer motivation in sub-Saharan Africa [29,31,33,34,36]. The findings paint a complex picture of the volunteers’ motivations for both entering and remaining in service. In relation to motivations for entering service, responses to both open- and close-ended questions highlight the importance of value-oriented functions and higher order social aspirations such as “helping society” or “humanity”. However, 70% of this study’s participants also agreed to at least one close-ended economic motivation statement and nearly a quarter (23%) agreed to all four. Illustrating economic need, as well as economic motivation, over half (53%) the study respondents agreed that they had become a volunteer because they needed help from the project. Volunteers with lower and mid-level SOL scores were significantly more likely to agree with economic motivation statements.

A number of studies globally have demonstrated the presence of economic motivations in volunteer cohorts [13,21,29,31,32,34,36,37,49-51]. This study, however, is one of the few in a low- or middle-income setting, which has explicitly sought to quantify the phenomenon and to relate it to volunteers’ current economic need. Such findings provide supportive evidence to combat the assumption that volunteers in low- and middle-income settings are sufficiently economically secure that their participation is wholly based on “unobligated free choice”. This is particularly critical in light of Maes’ [31] description of the way NGOs can exert pressure on volunteers to internalize self-sacrificing motivations, even in the face of requests for material compensation or reward.

Our large sample size enabled some assessment of the way volunteer motivations are influenced by gender and geography as well as by operational features of the programme. In this study, female volunteers scored significantly higher on the economic motivation index compared to men (*P* ˂ .001). Such findings possibly reflect the normative role of Zambian women as household food providers and their concurrent lack of control over household finances [52,53]. This may also provide a partial explanation for why Mama Waluse reported substantial material need despite having a comparatively higher SOL score. Another potential explanation is the higher opportunity cost that volunteering incurred for women.

Pointing to the higher cost of living in urban centres and the limited opportunities for subsistence farming, we also found that urban volunteers scored higher on the economic motivation index compared to rural volunteers (*P* ˂ .001). Although constrained by our limited capacity to assess programmatic features, our findings also mirror other work which has highlighted the importance of providing adequate supervision and educational or learning opportunities as a prerequisite to ensuring sustained and high-quality volunteer service [37,54]. However, further research is needed to better characterize the complex interplay between socio-economic status, socio-cultural norms, programme operations and volunteer motivations.

Reliance on uncompensated or minimally compensated voluntary labour has long been justified and defended as a “sustainable” – conflated with “low cost” – solution to weak health infrastructure and shortages in health personnel. While clearly demonstrating sacrificial and pro-social motivations, the common expression of volunteers’ own economic distress in this study points to the need for greater consideration of their material needs [31] and adds to the mounting evidence and ethical argument [13,20,31] against uncomplicated assumptions about volunteers’ circumstances. Indeed, earlier [18,55,56] and more recent [20,22,50] reviews of volunteer programmes have shown that successful integration of community health volunteers into national health systems requires substantial investment in worker selection, training, equipping and supervision, as well as proper remuneration. Recommendation #14 in the World Health Organizations’ [57] guidelines on task shifting similarly recognizes that the delivery of health services through voluntary labour is “not sustainable” and advises that adequate payment be made to community health workers. Our findings support such assertions and suggest that community health programmes dependent on community-source labour must give consideration to, and plan for, the economic needs and socio-political status of potential recruits.

Our mixed-method approach and the inclusion of the open-ended questions provided important insights, particularly in relation to the way Zambian volunteers’ frame economic distress. Situated in “local worlds” – their communities and the programme itself – that define volunteering and economic motivation as mutually exclusive, many respondents (including Mama Waluse) resorted to indirect modes of communication to convey their own need. Rather than a motive for entering service, for instance, volunteers’ own household vulnerability became *a barrier to serving others*; instead of securing livelihoods for themselves, requests for project support in income generation became *a means to provide for others*. As linguistic devices, these “idioms of [economic] distress” [58] permitted volunteers to articulate their own vulnerability while keeping the needs and interests of their clients in the foreground, as socially expected.

In addition to their own needs, volunteers in our sample consistently and freely described the economic distress of their clients. In replies related to motivations, barriers and expectations met and not met from the programme, volunteers made it abundantly clear that the provision of HIV-related psycho-social care or “health talk” were inadequate in a context of widespread material insecurity. Particularly vivid illustrations of the issue were found in individuals’ descriptions of negative encounters with “demanding” clients reacting to “empty handed” volunteers. Taken together, these findings help us see that this programme’s HIV focus resulted in falsely premised conceptualizations of both *help-giving* volunteers and of *help-needing* beneficiaries. Removing the obscuring filter of HIV brings into stark relief the more fundamentally important and common needs of volunteers and their clients alike: food, livelihoods, jobs and education for their children.

The use of mixed methods was a distinct strength of this study, helping us to move beyond a purely descriptive (quantitative or qualitative) analysis and enabling triangulation of the different types of data in order to check, interpret and refine our understanding of volunteer motivations [59,60]. Nonetheless, a number of limitations should be noted. Although designed to achieve a minimum level of representativeness, our participant recruitment was non-random. While every effort was made to ensure participants understood the scope and intention of the study, we cannot discount the possibility of self-selection bias among volunteers who perceived participation as a potential pathway to future employment. For these reasons, caution is required in generalizing our findings to other Zambian or regional volunteers. Our reliance on self-reported client numbers and the number of monthly visits may have led to over- or underestimation of volunteers’ commitment to service. Although somewhat mitigated by the triangulation with qualitative findings, dependence on dichotomous response options (versus more varied Likert scales) for the close-ended questions limited efforts to conduct a more sophisticated analysis. Simultaneous translation and transcription of participants’ short-answer responses to open-ended questions and the associated potential for mistranslation or other recording bias are acknowledged. While audio recording over 700 interactions was neither logistically nor financially feasible, the real-time quality assurance checks by multi-lingual supervisors effectively minimized this risk.

## Conclusions

This paper presents findings from a large opportunistic survey of volunteer HIV caregivers’ motivations across three districts in Zambia. Our findings demonstrate that volunteers had complex motivations for entering and continuing service, including “helping” and other pro-social values, but also manifest expectations of material or economic support. These findings contribute to evidence in support of various reforms needed to strengthen the viability and sustainability of volunteer-dependent health and community services in low- and middle-income settings. Primary among these are the need to do more to acknowledge and plan for the social and economic vulnerability of “volunteer” recruits.

## References

[1] World Health Organization (1978). Declaration of Alma Ata.

[2] Walt G (1988). Community health workers: are national programmes in crisis?. Health Policy Plan..

[3] Barker PM, McCannon CJ, Mehta N, Green C, Youngleson MS, Yarrow J (2007). Strategies for the scale-up of antiretroviral therapy in South Africa through health system optimization. J Infect Dis..

[4] Curan J, Curran J, Debas H, Arya M, Kelley P, Knobler S, and Pray L. editors. Scaling up treatment for the global AIDS pandemic: challenges and opportunities, Institute of Medicine (US) Committee on Examining the Probably Consequences of Alternative Patterns of Widespread Antiretroviral Drug Use in Resource-Constrained Settings. Washington (DC): National Academies Press; 2005. 25009882

[5] Larson E, O’Bra H, Brown JW, Mbengashe T, Klausner JD (2012). Supporting the massive scale-up of antiretroviral therapy: the evolution of PEPFAR-supported treatment facilities in South Africa, 2005–2009. BMC Public Health..

[6] Wagner G, Ryan G, Taylor S (2007). Formative evaluation of antiretroviral therapy scale-up efficiency in sub-Saharan Africa. AIDS Patient Care STDS.

[7] Estopinal CB, van Dijk JH, Sitali S, Stewart H, Davidson MA, Spurrier J (2012). Availability of volunteer-led home-based care system and baseline factors as predictors of clinical outcomes in HIV-infected patients in rural Zambia. PLoS One.

[8] Manara K. Civil society voluntarism in Tanzania. In: Kepa’s Working Papers. 2009

[9] Mieh TM, Iwelunmor J, Airhihenbuwa CO (2013). Home-based caregiving for people living with HIV/AIDS in South Africa. J Health Care Poor Underserved.

[10] Mutevedzi PC, Lessells RJ, Heller T, Bärnighausen T, Cooke GS, Newell ML (2010). Scale-up of a decentralized HIV treatment programme in rural KwaZulu-Natal, South Africa: does rapid expansion affect patient outcomes?. Bull World Health Organ.

[11] Rachlis B, Sodhi S, Burciul B, Orbinski J, Cheng AH, Cole D (2013). A taxonomy for community-based care programs focused on HIV/AIDS prevention, treatment, and care in resource-poor settings. Global Health Action..

[12] Sanjana P, Torpey K, Schwarzwalder A, Simumba C, Kasonde P, Nyirenda L (2009). Task-shifting HIV counselling and testing services in Zambia: the role of lay counsellors. Human Resources for Health..

[13] Wilson T. Incentives and volunteerism in Zambia: a review. In: Research partnerships build the service field in Africa. 2007. p. 68–84

[14] Campbell C, Nair Y, Maimane S (2007). Building contexts that support effective community responses to HIV/AIDS: a South African case study. Am J Community Psychol.

[15] Curran K, Njeuhmeli E, Mirelman A, Dickson K, Adamu T, Cherutich P (2011). Voluntary medical male circumcision: strategies for meeting the human resource needs of scale-up in southern and eastern Africa. PLoS Med.

[16] Jack BA, Kirton J, Birakurataki J, Merriman A (2011). ‘A bridge to the hospice’: the impact of a community volunteer programme in Uganda. Palliat Med..

[17] Ngongo Bahati P, Kidega W, Ogutu H, Odada J, Bender B, Fast P (2010). Ensuring quality of services in HIV prevention research settings: findings from a multi-center quality improvement pilot in East Africa. AIDS Care.

[18] Berman PA, Gwatkin DR, Burger SE (1987). Community-based health workers: head start or false start towards health for all?. Soc Sci Med.

[19] Morgan LM (2001). Community participation in health: perpetual allure, persistent challenge. Health Pol Plan.

[20] Akintola O (2008). Unpaid HIV/AIDS care in Southern Africa: forms, context, and implications. Feminist Econ.

[21] Celletti F, Wright A, Palen J, Frehywot S, Markus A, Greenberg A (2010). Can the deployment of community health workers for the delivery of HIV services represent an effective and sustainable response to health workforce shortages? Results of a multicountry study. AIDS..

[22] Hermann K, Van Damme W, Pariyo GW, Schouten E, Assefa Y, Cirera A (2009). Community health workers for ART in sub-Saharan Africa: learning from experience--capitalizing on new opportunities. Hum Res Health..

[23] Maes KC, Hadley C, Tesfaye F, Shifferaw S, Tesfaye YA (2009). Food insecurity among volunteer AIDS caregivers in Addis Ababa, Ethiopia was highly prevalent but buffered from the 2008 food crisis. J Nutr.

[24] Akintola O, Hlengwa WM, Dageid W (2013). Perceived stress and burnout among volunteer caregivers working in AIDS care in South Africa. J Adv Nurs.

[25] Omoto AM, Snyder M (1995). Sustained helping without obligation: motivation, longevity of service, and perceived attitude change among AIDS volunteers. J Pers Soc Psychol.

[26] Clary EG, Snyder M, Ridge RD, Copeland J, Stukas AA, Haugen J (1998). Understanding and assessing the motivations of volunteers: a functional analysis. J Pers Soc Psychol.

[27] Pinder CC (1998). Work motivation in organizational behavior, ed. U.S. River.

[28] Tremblay MA, Blanchard CM, Taylor S, Pelletier L, Villeneuve M (2009). Work extrinsic and intrinsic motivation scale: its value for organizational psychology research. Can J Behav Sci.

[29] Akintola O (2011). What motivates people to volunteer? The case of volunteer AIDS caregivers in faith-based organizations in KwaZulu-Natal, South Africa. Health Pol Plan..

[30] Kironde S, Klaasen S (2002). What motivates lay volunteers in high burden but resource-limited tuberculosis control programmes? Perceptions from the Northern Cape province, South Africa. Int J Tubercul Lung Dis.

[31] Maes K (2012). Volunteerism or labor exploitation? Harnessing the volunteer spirit to sustain AIDS treatment programs in urban Ethiopia. Hum Organ.

[32] Akintola O (2010). Perceptions of rewards among volunteer caregivers of people living with AIDS working in faith-based organizations in South Africa: a qualitative study. J Int AIDS Soc..

[33] Dil Y, Strachan D, Cairncross S, Korkor AS, Hill Z (2012). Motivations and challenges of community-based surveillance volunteers in the northern region of Ghana. J Comm Health..

[34] Greenspan JA, McMahon SA, Chebet JJ, Mpunga M, Urassa DP, Winch PJ (2013). Sources of community health worker motivation: qualitative study in Morogoro Region, Tanzania. Hum Res Health..

[35] Olang’oa CO, Nyamongoa IK, Aagaard-Hansenb J (2010). Staff attrition among community health workers in home-based care programmes for people living with HIV and AIDS in western Kenya. Health Pol.

[36] Maes K, Kalofonos I (2013). Becoming and remaining community health workers: perspectives from Ethiopia and Mozambique. Soc Sci Med..

[37] Schneider H, Hlophe H, van Rensburg D (2008). Community health workers and the response to HIV/AIDS in South Africa: tensions and prospects. Health Policy Plan.

[38] World Bank. Zambia country profile. 2014; Available from: http://www.worldbank.org/en/country/zambia/overview (viewed 17 Oct 2015).

[39] Central Statistical Office and Ministry of Health (2009). Zambia demographic and health survey 2007.

[40] MOH, GRZ (2011). Annual health statistical bulletin 2010.

[41] US Department of State (2010). Leading through civilian power: the first quadrennial diplomacy and development review.

[42] Ryan RM, Deci EL (2000). Intrinsic and extrinsic motivations: classic definitions and new directions. Contemp Educ Psychol..

[43] Leech NL, Onwuegbuzie AJ (2009). A typology of mixed methods research designs. Qual Quan..

[44] Bowden A, Fox-Rushby JA (2003). A systematic and critical review of the process of translation and adaptation of generic health-related quality of life measures in Africa, Asia, Eastern Europe, the Middle East, South America. Soc Sci Med..

[45] Kirkpatrick P, van Teijlingen E (2009). Lost in translation: reflecting on a model to reduce translation and interpretation bias. Open Nurs J..

[46] Montgomery M, Gragnolati M, Burke KA, Paredes E (2000). Measuring living standards with proxy variables. Demography..

[47] Onwuegbuzie AJ, Combs JP, Tashakkori A, Teddlie C (2010). Emergent data analysis techniques in mixed methods research: a synthesis. SAGE handbook of mixed methods in social and behavioral research.

[48] Sandelowski M (2001). Real qualitative researchers do not count: the use of numbers in qualitative research. Res Nurs Health..

[49] Ashraf, Nava, and Kindred Natalie. Community Health Workers in Zambia: Incentive Design and Management. Harvard Business School Case 910–030, March 2010. (Revised February 2014).

[50] Lehmann U, Sanders D (2007). Community health workers: what do we know about them? The state of the evidence on programmes, activities, costs and impact on health outcomes of using community health workers.

[51] Sunkutu K, Nampanya-Serpell N (2009). Searching for common ground on incentive packages for community workers and volunteers in Zambia.

[52] Doss CR. Twenty-five years of research on women farmers in Africa: lessons and implications for agricultural institutions, in Economics Program Paper No.99-02. 1999: Mexico.

[53] Farnworth CR, Munachonga M (2010). Gender approaches in agricultural programmes - Zambia country report: a special study of the Agricultural Support Programme (ASP). UTV Working Paper 2010:8.

[54] Maes K, Closser S, Kalofonos I (2014). Listening to community health workers: how ethnographic research can inform positive relationships among community health workers, health institutions, and communities. Am J Public Health.

[55] Fendall R (1984). Discussion: we expect too much from community health workers. World Health Forum..

[56] Skeet M (1984). Community health workers: promoters or inhibitors of primary health care?. World Health Forum..

[57] World Health Organization (2008). Task shifting: rational redistribution of tasks among health workforce teams.

[58] Nicther M (2010). Idioms of distress revisited. Cult Med Psychiatr..

[59] Chen H (1997). Applying mixed methods under the framework of theory-driven evaluation. New Dir Eval..

[60] Caracelli V, Green J (1997). Crafting mixed-method evaluation design. New Dir Eval..

